# Comparative Susceptibility Study Against Pathogens Using Fermented Cranberry Juice and Antibiotics

**DOI:** 10.3389/fmicb.2019.01294

**Published:** 2019-06-07

**Authors:** Ioanna Mantzourani, Christos A. Bontsidis, Stavros Plessas, Athanasios Alexopoulos, Eirini Theodoridou, Christina Tsigalou, Chrysa Voidarou, George Douganiotis, Stavros L. Kazakos, Elisavet Stavropoulou, Eugenia Bezirtzoglou

**Affiliations:** ^1^Laboratory of Microbiology, Biotechnology and Hygiene, Faculty of Agriculture Development, Democritus University of Thrace, Orestiada, Greece; ^2^Department of Agriculture, Faculty of Agriculture, Food Science, Nutrition, University of Ioannina, Ioannina, Greece; ^3^Laboratory of Microbiology, Medical School, Democritus University of Thrace, Alexandroupolis, Greece; ^4^Theageneio Cancer Hospital, 3rd Department of Clinical Oncology, Thessaloniki, Greece; ^5^Service de Medicine Interne, Centre Hospitalier Universitaire Vaudois, Lausanne, Switzerland

**Keywords:** antibiotics, pathogens, probiotic, cranberry juice, fermentation

## Abstract

In the present study, unfermented and fermented cranberry juice in combination with the Antibiotics vancomycin and tigecycline were tested for their antimicrobial activity. Cranberry juice was fermented with a recently isolated potentially probiotic *Lactobacillus paracasei* K5. The tested strains selected for this purpose were *Enterococcus faecalis, E. faecium, Enterobacter cloacae* and *Staphylococcus aureus*. The methods followed were the determination of zones inhibition, Minimum Inhibitory Concentration (MIC) and Fractional Inhibitory Concentration Index (FICI). Tigecycline together with fermented juice exhibited larger Zones of Inhibition (ZOI) in strains of *E. faecium* (65 ± 4.8 mm) compared to the respective ZOI with tigecycline and unfermented juice (no zone). The same outcome was also obtained with *E. cloacae*. Vancomycin together with fermented juice exhibited larger ZOI in strains of *E. faecium* (28 ± 2.2 mm) compared to the respective ZOI with vancomycin and unfermented juice (24 ± 2.3 mm). The lowest MIC values were recorded when tigecycline was combined with fermented cranberry juice against *S. aureus* strains, followed by the same combination of juice and antibiotic against *E. cloacae* strains. FICI revealed synergistic effects between fermented juice and tigecycline against a strain of *E. faecium* (A2020) and a strain of *E. faecalis* (A1940). Such effects were also observed in the case of fermented juice in combination with vancomycin against a strain of *S. aureus* (S18), as well as between fermented juice and tigecycline against *E. cloacae* (E1005 and E1007) strains. The results indicate that the antibacterial activity of juice fermented with the potentially probiotic *L. paracasei* K5 may be due to synergistic effects between some end fermentation products and the antibiotic agents examined.

## Introduction

Cranberries are perennial plants, with characteristic reddish color, which belong to section *Cyanococcus*, genus *Vaccinum.* The greater family is *Ericaceae*, a family of edible fruits which also includes blueberries and gooseberries. These fruits are rich in phenolics (flavonoids, flavonols, catehins, and anthocyanins), organic acids (citric, malic, benzoic, and ferulic), and, in the case of cranberries, also proanthocyanins – a special group of condensed tannins. Cranberries are cultivated in Central and Northern Europe, and also Canada and Chile (Vattem et al., 2005). They first came into the spotlight because of their biological properties and health benefits to consumers.

Lactic acid bacteria (LAB) belong to a wide bacteria family whose fermented activity ends with lactic acid. They include species such as *Lactobacillus. plantarum, L. sakei, L. casei, L. paracasei, L. fermentum, Leuconostoc mesenteroides, Weissela confuse, W. cibaria, Pediococcus pentosaceus* (Todorov et al., 2008). These strains are used in several traditional food fermentation methods for liquid state fermentation (LSF) with plant- based substrates. The main reason for their use is the belief that they induce health benefits, including the production of bioactive compounds, as well as possess antimicrobial, antiinflammatory, and antioxidant (Liu et al., 2011; Liao et al., 2013; Torino et al., 2013; Limon et al., 2015), and antiobesity (Lee et al., 2013) properties (Muhammed et al., 2018).

The microenvironment within fruit juices favors LAB fermentation, due to availability of minerals and vitamins, and an acidic pH (2,4–3,5) (Kim et al., 2014; Swain et al., 2014). During lactic fermentation of fruit juices, flavonoids are released, resulting in high antioxidative activity. With the application of suitable strains, a large variety of enzymes, such as glucosidases, amylases, celluloses, xylanases, esterases, invertases and lipases, alter the structure of phytochemical compounds present in fruit juices. LAB contribute to the degradation of high molecular weight phenolic compounds (Othman et al., 2009; Hur et al., 2014). Supplementary factors, contributing to microbial inhibition and antioxidative activity of fermentation products by LAB are: low pH, hydrogen peroxide, oxygen, temperature, time and extraction and bacteriocins (Kantachote et al., 2008). In our study, the probiotic strain *Lactocacilus paracasei* K5, previously characterized in our laboratory (Plessas et al., 2017), was used for the fermentation of cranberry juice.

*Lactobacillus paracasei* exists as part of the normal flora of the human gastrointestinal tract. Naturally fermented vegetables, milk, and meat may also contain strains of *L. paracasei* (Swain et al., 2014). The strain used in the present study was a wild one isolated in our laboratory that exhibited antioxidant, probiotic, and antiproliferative properties (Chondrou et al., 2018). For this reason, it was selected in order to examine the properties of a fermented cranberry juice compared to an unfermented one.

Natural products, like cinnamon extracts, essential oils, and their compounds may inhibit bacteria by various methods, such as damaging the cell membrane, altering the lipid profile, inhibiting ATPases, cell division, membrane porins, motility, and biofilm formation (Vasconcelos et al., 2018). Particularly for cranberry, a study has shown to prevent urinary tract infections by interfering with bacterial adhesion in the urinary tract (Howell et al., 1998). Uropathogenic *Escherichia coli* strains are not able to adhere to bladder cell receptors (Howell et al., 2010) and thus, cell growth is inhibited and infection is prevented. It has been speculated that the observed reduction in adhesion may be due to changes in bacterial morphology (Liu et al., 2006) and/or genetically based decreases in P-fimbrial expression (Ahuja et al., 1998; Liu et al., 2006). In another study, antiadherence properties were demonstrated when cranberry extracts were applied against pathogens. These effects have been associated with cranberry juice inhibiting *E. coli* adherence to uroepithelium and its subsequent multiplication (Blumberg et al., 2013; Jensen et al., 2017; Kim et al., 2019).

Antibiotic agents are chemical substances derived from bacteria or fungi that inhibit the growth of other strains. The basic mode of inhibition includes blocking basic cellular procedures, like cell wall synthesis, transcription, translation, energy production, etc. In the present study, two broad-spectrum antibiotic substances, vancomycin and tigecycline, were tested for their activity against pathogenic strains. Vancomycin is a glycopeptide antibiotic which acts by inhibiting cell wall synthesis and is used for the treatment of Gram-positive bacteria. Tigecycline is a tetracycline antibiotic which acts by inhibiting protein synthesis and is used against both Gram- negative and Gram-positive bacteria. In our study, vancomycin was used against *Staphylococcus aureus* strains and tigecycline was selected mostly against *Enterococcus* spp. and *Enterobacter* spp. based on the results of a previous study in our laboratory (Mantzourani et al., 2015).

*Enterococcus* spp. are commonly responsible for severe infections of the urinary tract, septicemia and meningitis. Especially *E. faecium* has been a leading cause of multi-drug resistant enterococcal infections. *E.* spp. are commonly responsible for infections of the urinary and respiratory tracts (Fisher and Phillips, 2009). *S. aureus* can cause pneumonia, meningitis, bacteremia and sepsis. It should be noted that *S. aureus* strains are part of the normal skin flora and are normal inhabitants of the lower reproductive tract of women.

In the present study, the antimicrobial activity of cranberry juice, both unfermented and fermented with *L. paracasei* K5 (Plessas et al., 2017), was tested against several pathogens, like *S. aureus, E. faecium* and *E. faecalis* and *E. cloacae*. The results were compared with the action of two antibiotic agents, Vancomycin and Tigecycline against the same pathogens. The Fractional Inhibitory Concentration Index (FICI) was calculated in each case in order to examine synergism or antagonism of their bioactive components.

The goal of the study was to investigate whether or not fermented cranberry juice and commercial antibiotics can be used in combination as an alternative treatment to multi-drug resistant bacterial infections, by pinpointing the decrease of the minimum inhibitory concentration of these severe pathogens with the simultaneous consumption of cranberry juice.

## Materials and Methods

### Cranberry Fruit Preparation for Fermentation

Ten kilograms of fresh cranberry fruits were obtained directly from a farm in Marasia, north-eastern Greece. The fresh fruits were carefully selected, washed, the kernel was removed, and the fruits processed into juice by blending for 10 min in a common household blender. The final product was added into deionized water (1:2 proportion) and the mixture was submitted to blanching at 90°C constant temperature for 2 min. The remaining juice was extracted by cloth filtration and pasteurized at 80°C for 5 min. The initial value of pH was adjusted to 3.5 through the addition of NaOH 0.1 M. A dilution of *L. paracasei K5* with turbidity of 0.5 McFarland scale was added in 100 mL of fermentation substrates. The final concentration of the probiotic strain was 2% w/v, viability approximately 10^8^ CFU/mL. The unfermented samples were incubated for 24 h at 30°C for fermentation. The fermentations were carried out in triplicate. The fermented and the unfermented juice samples were tested for their antimicrobial activity against pathogens: *S. aureus, E. faecium, E. Faecalis*, and *E. cloacae*.

### Microbiological Analysis

Fermented and unfermented juice samples were screened for their antibacterial activity, according to the agar well diffusion method proposed by the CLSI (former NCCLS) against the following nosocomial pathogens: *S. aureus, E*. *faecium, E. faecalis* and *E. cloacae*.

All the above bacteria were cultivated in Trypticase Soy Broth (TSB) at 37°C for 18 h and purified on blood agar plates (Merck Co., United States) supplemented with 5% sterile defibrinated sheep’s blood.

The antibiograms were conducted on Muller Hinton Agar (M173-500G HIMEDIA, India) with the disk diffusion method. Accordingly, a 10 μL aliquot of each juice sample was added on sterile disks and cultures were incubated at 37°C for 18 h. Antibacterial activity was assessed by measuring (with a caliper) the diameter of the inhibition zones surrounding the disks. The same process was followed for the evaluation of the antimicrobial activity of two antibiotics against pathogens. The antibiotics examined were Vancomycin (30 μg/disk HIMEDIA) and Tigecycline (15 μg/disk HIMEDIA). The inoculated plates were allowed to stand at room temperature for 15 min prior to dispensing the paper disks and the plates incubated at 37°C for 24 h. The diameters of the clear zones around each disk were measured after incubation.

During the microdilution method, antimicrobial drugs were tested in the range of 0.5–512 μg/mL for vancomycin, in the range of 0.002–2 μg/mL for tigecycline and the fermented and the unfermented juice in the range of 1–1024 μg/mL. The above concentrations were chosen based on analogous published reports (Wojnicz et al., 2016), to be comparable with the concentration of antibiotics and to be at least four times above/ below the MIC for the estimation of FIC values. A control test was performed without any antimicrobial agent. MIC was defined as the lowest concentration of antimicrobial agent that inhibited the visible growth of test organism. Each experiment was conducted in triplicate.

### Determination of FIC and FIC Index (FICI)

The combined effect of the two antibiotics and the fermented – unfermented juice (FIC) was evaluated by the microdilution chequerboard method with some modifications (Fratini et al., 2017). In the first of a series of six test tubes each containing 20 mL Mueller-Hinton broth, an amount of 163.84 μL fermented or unfermented juice was added (equal to a concentration of 8192 μg/mL or eight times the estimated MIC). Then, 10 mL from the first tube was serially diluted up to the sixth tube. The final 10 mL were discarded. Test tubes with antibiotics (tigecycline and vancomycin) were prepared accordingly. 160 μL from each tube with cranberry juice were transferred to the first, second, third, etc., column of a 96 micro-well plate. An equal amount (160 μL) from the test tubes containing the antibiotics were added to the first, second, third, etc., row of the same micro-plate. In this manner the top-left well was contained a combination of four times the MIC of juice and four times the MIC of antibiotic followed by a series of dilutions of the above in order to obtain the FIC final concentrations (4 MIC0, 2 MIC0, 1 MIC0, MIC0/2, MIC0/4, and MIC0/8). Finally, 10 μL of bacterial suspension standardized at 0.5 McFarland standard turbidity units were added in each well, except the negative control, in which 10 μL of sterile BHI (Brain Heart Infusion) broth was added. Microplates were incubated at 37°C for 24 h. FIC determinations were performed in triplicate. For each experiment, FICI values were calculated using the following formula:

FICI=FICjuice+FICantibiotic

where FICjuice = MICjuice in combination/MICjuice alone and FICantibiotic = MICantibiotic in combination/MICantibiotic alone.

According to EUCAST (2000) a Synergistic effect is observed when FICI value ≤ 0.5; an Additive effect when 0.5 < FICI value ≤ 1; an Indifferent effect when 1 < FICI value < 2 and an Antagonistic effect when FICI value ≥ 2.

### Statistical Analysis

Inhibition zones (mm) of fermented and unfermented cranberry juice were compared for any difference of the mean by using the Student *t*-test at a significance level of 0.05. Inhibition zones among the two antibiotics with fermented and unfermented cranberry juice were compared by using the ANOVA procedure with Tukey’s HSD *post hoc* comparison. All analyses were performed with IBM SPSS Statistics for Windows, Version 20.0 (Armonk, NY, United States: IBM Corp).

## Results

### Antimicrobial Activity With Zones Inhibition Method

The zones of inhibition (ZOI) were measured following the application of cranberry juice, both unfermented and fermented with *L. paracasei* K5. The concentration of unfermented cranberry juice used was 1000 mg/mL while that of fermented juice was the same after 24 h of fermentation with the addition of *L. paracasei* K5 2% w/v (turbidity 10^8^ CFU/mL). The results are summarized in Table 1.

**Table 1:** Inhibition zones for 10 μL unfermented and 10 μL fermented cranberry juice (1000 mg/mL+2% *L. paracasei*) alone against *Enterococcus faecium, E. faecalis, Enterobacter cloacae* and *S. aureus*.

Isolate	Unfermented cranberry juice ZOI	Fermented cranberry juice ZOI
	**(mm)**	**(mm)**

***Enterococcus faecium***		
A 1337	No zone	No zone
A 1658	No zone	No zone
A 1668	No zone^a^	8.25 ± 0.5^b^
A 2020	10.45 ± 1.1^a^	10.75 ± 1.2^a^
A 1709	10.55 ± 1.4^a^	10.70 ± 1.2^a^
***Enterococcus. faecalis***		
A 1931	No zone	No zone
A 1957	No zone	No zone
A 1940	No zone	No zone
A 1951	17 ± 1.6^a^	17.75 ± 2.1^a^
A 1966	15 ± 1.8^a^	15.45 ± 1.8^a^
***Enterobacter cloacae***		
E 1000	No zone	No zone
E 1002	No zone	No zone
E 1004	No zone	No zone
E 1005	13 ± 1.1^a^	14 ± 1.8^a^
E 1007	11 ± 0.5^a^	13 ± 1.5^b^
***S. aureus***		
S 1530	No zone	No zone
S 15	No zone	No zone
S 12	No zone	No zone
S 18	No zone^a^	8.2 ± 0.4^b^
S 16	No zone^a^	9.5 ± 1.0^b^


*Different superscript letters indicate a statistically significant difference between the two means (Student’s *t*-test).*

In the case of *E. faecium*, the A2020 and A1707 strains exhibited the largest ZOI with fermented and the unfermented juice (10.75 ± 1.2, 10.45 ± 1.1 mm and 10.70 ± 1.2, 10.55 ± 1.4 mm, respectively). *E. faecalis* strains A1951 and A1966 showed the greatest ZOI ranging from 17.75 ± 2.1 to 17 ± 1.6 mm and 15 ± 1.8 to 15.45 ± 1.8 mm with fermented and the unfermented juice samples, respectively. *S. aureus* strains displayed the lowest values of inhibition zones, since their diameter reached 8.2 ± 0.4 and 9.5 ± 1.0 mm for the strains S18 and S16, respectively, and only in the case the fermented juice samples.

*Enterobacter cloacae* strains E1005 and E1007 showed enlarged ZOI, 14 ± 1.8 and 13 ± 1.5 mm, respectively, compared to those of the unfermented juice samples (13 ± 1.1 and 11 ± 0.5 mm for the same strains).

**FIGURE 1:**
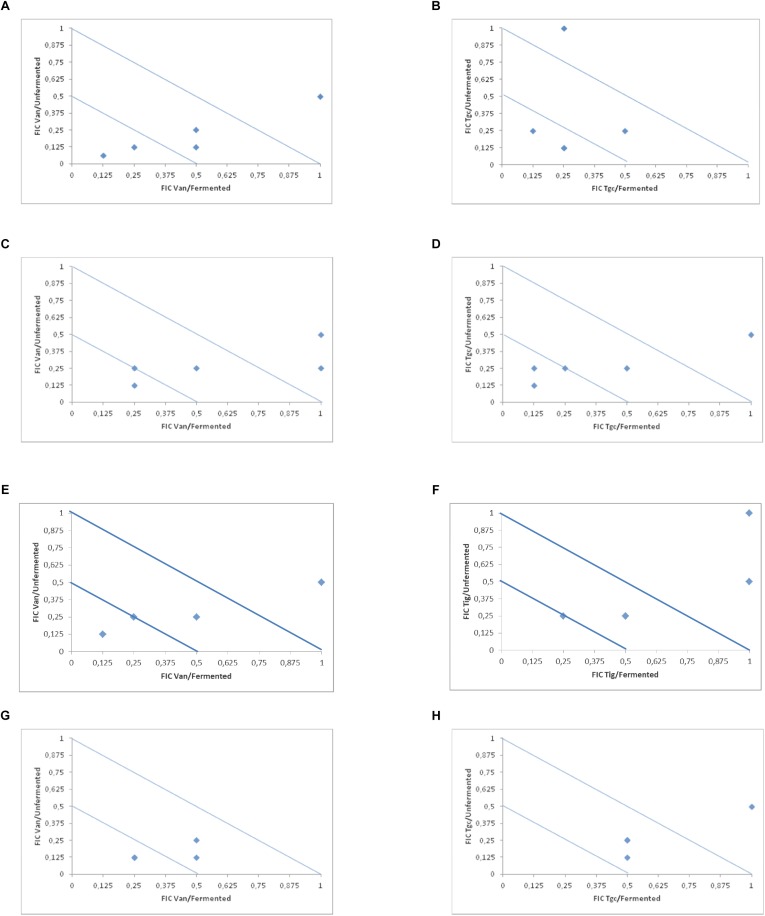
Isobolograms of *in vitro* drugs and fermented and unfermented cranberry juice (vancomycin and tigecycline) against *E. faecalis* **(A**,**B)**, *E. faecium* **(C**,**D)**, *E. cloacae* **(E**,**F)** and *S. aureus* **(G**,**H)**.

In Table 2 and Figure 1 (isobolograms), the results of the combined activity of the two antibiotic agents and fermented and the unfermented cornelian cherry juice are presented.

**Table 2:** Inhibition zones for 10 μL unfermented and 10 μL fermented cranberry juice (1000 mg/mL+2% *L. paracasei*) in combination with Vancomycin and Tigecycline (15 μg/disk) against *Enterococcus faecium, E. faecalis, Enterobacter cloacae* and *S. aureus*.

Isolate	Vancomycin + Unfermented cranberry juice ZOI	Vancomycin + Fermented cranberry juice ZOI	Tigecycline + Unfermented cranberry juice ZOI	Tigecycline + Fermented cranberry juice ZOI	Vancomycin ZOI	Tigecycline ZOI
	**(mm)**	**(mm)**	**(mm)**	**(mm)**	**(mm)**	**(mm)**

***E*nterococcus *faecium***						
A 1337	24 ± 2.3^a^	28 ± 2.2^a^	No zone	65 ± 4.8^c^	18 ± 3	42 ± 7
A 1658	No zone	23 ± 2.6^a^	11 ± 1.2^b^	29 ± 2.8^c^	16 ± 2.2	25 ± 4.3
A 1668	15 ± 1.5^a^	20 ± 2.1^a^	28 ± 2.2^b^	32 ± 3.1^b^	15 ± 2.5	22 ± 3.6
A 2020	16 ± 1.9^a^	19 ± 1.5^a^	34 ± 3.1^b^	60 ± 4.0^c^	14 ± 2.1	45 ± 4.8
A 1709	No zone	24 ± 2.4^a^	12 ± 1.8^b^	46 ± 2.1^c^	12 ± 1.1	30 ± 3.8
***E*nterococcus *faecalis***						
A 1931	No zone	19 ± 2.0^a^	No zone	30 ± 3.9^b^	14 ± 2.8	14 ± 2.2
A 1957	No zone	24 ± 2.5^a^	No zone	38 ± 2.1^b^	15 ± 2.6	35 ± 3.5
A 1940	No zone	No zone	No zone	15 ± 1.2	10 ± 1.4	10 ± 1.2
A 1951	No zone	No zone	10 ± 0.5^a^	16 ± 2.2^b^	8 ± 0.8	12 ± 1.2
A 1966	14 ± 3.1^a^	20 ± 3.1^b^	15 ± 1.5^a^	20 ± 2.6^b^	11 ± 1.2	13 ± 2.1
***E*nterobacter *cloacae***						
E 1000	No zone	25 ± 1.4^a^	8 ± 1.1^b^	50 ± 4.1^c^	–	25 ± 2.7
E 1002	11 ± 1.0^a^	14 ± 2.0^b^	28 ± 3.3^c^	28 ± 2.2^c^	–	20 ± 1.8
E 1004	10 ± 1.1^a^	16 ± 2.4^b^	No zone	30 ± 2.6^c^	–	21 ± 1.5
E 1005	No zone	22 ± 2.7^a^	No zone	40 ± 3.3^b^	–	29 ± 2.4
E 1007	11 ± 1.1^a^	17 ± 2.0^b^	No zone	34 ± 2.8^c^	–	30 ± 3.4
**S. aureus**						
S 1530	No zone	No zone	No zone	No zone	–	10 ± 1.1
S 15	No zone	No zone	No zone	8 ± 0.3	–	–
S 12	No zone	No zone	10 ± 1.8^a^	14 ± 2.2^b^	12 ± 2.3	10 ± 1.7
S 18	No zone	8.2 ± 2.3^a^	8 ± 1.5^a^	11 ± 1.6^a^	4 ± 0.5	4 ± 0.4
S 16	No zone	9.5 ± 2.1^a^	9 ± 1.0^a^	11 ± 1.6^b^	5 ± 0.8	5 ± 0.5


*Different superscript letters in a row indicate statistically significant differences (ANOVA with Tukey’s HSD *post hoc* comparisons).*

As far as the Gram-positive strains are concerned, the greatest ZOI were recorded following the application of tigecycline in combination with fermented juice against *E. faecium* strains with a diameter of 65 ± 4.8 and 60 ± 4.0 mm for the strains A1337 and A2020. The combination of vancomycin and fermented or unfermented juice exhibited similar ZOI, however, the fermented juice samples and vancomycin provided even larger zones compared to the unfermented juice.

In the case of *E. faecalis*, strains A1931 and A1957 displayed the greatest ZOI when treated with fermented cranberry juice in combination with tigecycline, i.e., 30 ± 3.9 and 38 ± 2.1 mm, respectively.

*Staphylococcus aureus* strains were the most resistant, even when tested with tigecycline and fermented cranberry juice. The strain S12 reached a diameter of 14 ± 2.2 mm with the same combination. Vancomycin does not seem to have antimicrobial activity against these *S. aureus* strains.

Finally, *E. cloacae* strains provided significantly large ZOI in both cases, i.e., tigecycline and fermented juice or vancomycin and tigecycline. The former combination in strains E1000 and E1005 showed ZOI of diameters 50 ± 4.1 and 40 ± 3.3 mm, respectively.

### Determination of Antimicrobial Activity With MIC Method

Minimum inhibitory concentration was defined as the lowest concentration of antibiotic (vancomycin, tigecycline), fermented and unfermented cranberry juice and their combination inhibiting visible growth of tested strains.

The lowest MIC values were recorded for tigecycline against *S. aureus* strains, varying from 0.004 to 0.032 μg/mL, whereas in the case of vancomycin, MIC values ranged from 16 to 32 μg/mL for all the strains.

Fermented juice showed lower MIC values compared to the unfermented one, in the majority of the pathogens tested, reaching a value of 512 μg/mL in contrast to the unfermented cranberry juice, where MIC a value 1024 μg/mL appeared in most of the strains tested.

When the combination of antibiotics and juice was tested, MIC values were significantly lower. In particular, vancomycin and fermented juice reached MIC values ranging from 2 μg/mL (in strains A1658, E1007, S18) to 32 μg/mL (strain A1966). In comparison with MIC values from the combined activity of vancomycin and unfermented juice, the range was the same (2–32 μg/mL), but in each strain, the respective MIC values were higher with the fermented juice.

Minimum Inhibitory Concentration values with tigecycline were even lower, ranging from 0.002 to 0.064 μg/mL in combination with the fermented juice, and from 0.008 to 0.128 μg/mL in combination with the unfermented juice. *S. aureus* strains were the most sensitive strains followed by the *E. cloacae* strains.

In conclusion, tigecycline and fermented cornelian cherry juice was the most effective combination against the majority of pathogens tested.

At this point, it should be stated that the pH of the samples of the fermented and unfermented cranberry juice was adjusted to 3.5–3.6 to ensure that the results would not be confounded by their difference in acidity, and because a pH value of 3.6 is the optimum value for *L. paracasei*. This conclusion was reached in a previous study in our laboratory, in which the chemical composition of cranberry juice fermented with *L. paracasei* was determined at different pH values (Nouska et al., 2016). The total sugar concentration in the unfermented cranberry juice was 55 g/L while the respective value in the fermented cranberry juice after 24 h of fermentation was 25.33 g/L.

### Determination of FICI

In Table 3, we observed that FICI = 0.5, which reveals synergism, appeared in strain A2020 (*E. faecium*) when tigecycline and fermented cranberry juice is applied. Among *E. cloacae* strains, E1004, E1005, and E1007, exhibited FICI 0.5, 0.5, and 0.25, respectively, in the case of vancomycin.

**Table 3:** MIC, FIC and FIC index (FICI) of fermented and unfermented cranberry juice with vancomycin and tigecycline by the microdilution method against pathogens.

	Minimum Inhibitory Concentration (μg/mL)								Fractional Inhibitory Concentration				FIC Index	
**Isolate**	**Van**	**Tig**	**Unfermented cranberry juice**	**Fermented cranberry juice**	**Van + Unfermented cranberry juice**	**Van + Fermented cranberry juice + Van**	**Tig + Unfermented cranberry juice**	**Tig+ Fermented cranberry juice**	**Van/Unf**	**Van/Fer**	**Tig/Unf**	**Tig/Fer**	**Van**	**Tig**

***Enterococcus faecium***														
A 1337	32	0,128	1024	512	16	8	0,128	0,064	0,5	0,25	1	0,5	0,75	1,5
A 1658	16	0,064	512	512	8	2	0,032	0,016	0,5	0,125	0,5	0,25	0,625	0,75
A 1668	16	0,128	1024	512	16	8	0,128	0,032	1	0,5	1	0,25	1,5	1,25
A 2020	16	0,128	1024	1024	16	8	0,032	0,032	1	0,5	0,25	0,25	1,5	**0,5**
A 1709	32	0,128	512	512	32	8	0,128	0,032	1	0,25	1	0,25	1,25	1,25
***Enterococcus faecalis***														
A 1931	32	0,128	1024	1024	16	8	0,128	0,032	0,5	0,25	1	0,25	0,75	1,25
A 1957	32	0,016	512	512	16	8	0,008	0,008	0,5	0,25	0,5	0,5	0,75	1
A 1940	16	0,128	1024	512	4	16	0,064	0,032	0,25	1	0,5	0,25	1,25	0,75
A 1951	16	0,128	512	512	16	8	0,128	0,016	1	0,5	1	0,125	1,5	1,125
A 1966	16	0,128	1024	1024	16	32	0,128	0,064	1	2	1	0,5	3	1,5
***Enterobacter cloacae***														
E 1000	16	0,032	1024	1024	16	8	0,032	0,032	1	0,5	1	1	1,5	2
E 1002	16	0,064	1024	1024	8	4	0,032	0,016	0,5	0,25	0,5	0,25	0,75	0,75
E 1004	16	0,064	1024	1024	4	4	0,032	0,016	0,5	0,25	0,5	0,25	**0,75**	0,75
E 1005	16	0,008	1024	1024	4	4	0,002	0,002	0,5	0,25	0,25	0,25	**0,75**	0,5
E 1007	16	0,064	1024	1024	2	2	0,016	0,016	0,5	0,125	0,25	0,25	**0,625**	**0,5**
***S. aureus***														
S 1530	16	0,032	512	1024	16	4	0,032	0,032	1	0,25	1	1	1,25	2
S 15	16	0,004	512	1024	8	4	0,004	0,002	0,5	0,25	1	0,5	0,75	1,5
S 12	16	0,016	512	1024	8	4	0,016	0,004	0,5	0,25	1	0,25	0,75	1,25
S 18	16	0,016	512	1024	4	2	0,008	0,004	0,25	0,125	0,5	0,25	**0,375**	0,75


Among *S. aureus* strains, only S18 had an FICI = 0.375, and only in the case of vancomycin. In most of the strains examined, FICI revealed synergism or additivity. In few cases, we observed indifferent effect or even antagonism between antibiotic agents and cranberry juice. *E. faecium* included the greatest percentage of strains against which a synergistic interaction between juice and antibiotics was observed.

## Discussion

In this study, the antimicrobial activity of cornelian cherry juice, unfermented or fermented with the probiotic *L. paracasei* K5, was examined against Gram-positive bacteria (*S. aureus - – E. Faecium – - E. faecalis*) and Gram-negative bacteria (*E. cloacae*). In addition, the synergistic interaction of the fermented and unfermented juice and two antibiotic agents, vancomycin and tigecycline was determined.

The results support the hypothesis that phenolic compounds present in cranberry juice might contribute to an enhanced antimicrobial effect. Lacombe et al. (2012) reported that berries extracts could inhibit pathogens and help probiotic strains compete with them for the adhesion to the intestinal wall. In another study, the fermented cranberry juice was tested for its antimicrobial activity against *Listeria monocytogenes, Vibrio parahaemolyticus*, and *E. coli O157*:*H7*. The results implied that the increased levels of gallic acid after fermentation resulted in increased antibacterial activity (Vattem et al., 2004). In addition, when the interaction between the pathogens *Mycobacterium tuberculosis* and *Penicillium avellaneum* and Chinese herbal medicines was investigated, enhanced antibacterial activity of the fermented products of certain herbs was proved (Yu-Ling et al., 2013).

Phytochemicals, like tannins, and products which derive from their hydrolysis, like ellagic acid, are able to inhibit the growth of microorganisms. This inhibition takes place mostly by blocking the basic functions of the bacterial membrane (ion channels and proteolytic activity) (Muhammed et al., 2018). Proteins sensitive to pH and ionic strength may be inactivated from the phenolic induced acidity.

The mechanism that may explain the enlarged ZOI we observed when fermented cranberry juice was used against pathogens is the free radical quenching capacity that phenolic compounds have. More specifically, the ability of phenols to cause hyperacidification, alter the permeability of the transmembrane and protein ion-channels activating many signaling pathways to eukaryotes and prokaryotes. Studies have shown that methylated phenolic compounds have excellent antibacterial properties against Gram-positive bacteria (Osbourn, 1999).

The lower MIC values were recorded following the application of tigecycline and fermented juice, especially against *S. aureus* strains (0,002–0,008 μg/mL). The combination of vancomycin and fermented juice resulted in the lowest MIC values against *S. aureus* strains, as well. This synergistic effect is in accordance with cranberry synergies also recorded with other functional phytochemicals and fruit extracts.

Wojnicz et al. (2016), examined the impact of cranberry juice on the virulence factors and biofilm formation by *E. faecalis* strains isolated from urinary tract infections. Their results indicate that cranberry extract reduces survival of these strains, mostly because of the high level of polyphenols that generate hydrogen peroxide and alter the permeability of the cell membrane.

Microorganisms stressed by exposure to polyphenols upregulate defensive proteins and downregulate various metabolic proteins (Ferrazzano et al., 2011). This fact may explain the antagonism exhibited in *E. faecalis* strains between cranberry juice and antibiotics (Yamanaka-Okada et al., 2008).

Our results are moreover in accordance with a nosocomial study (Bonetta et al., 2017), where a total of 924 patients with prostate carcinoma were randomized to receive or not enteric-coated cranberry extract for 6–7 weeks. Among the pathogens recorded were *E. faecalis* and *E. cloacae* strains. The outcome demonstrated that the treatment with enteric-coated cranberry extract could lessen the actinic damage to the bladder mucosa and reduce the inflammatory process, while the use of cranberry was shown to reduce the necessary antibiotic dose by 50%, a clear indication of synergistic activity between cranberry and antibiotics.

Synergy can be defined as the ability of two or more functional components to mutually enhance their functionalities, so that when they are present together, their combined function rapidly improves the overall result of maintaining the homeostasis in eukaryotes or killing pathogenic bacteria. An example of this action is the co-administration of coffee with ellagic acid (Abraham, 1996) or even cranberry phenolics with N-methyl-N-nitroso-guanidine (MNNG) that showed increased antimutagenic functionality (Vattem et al., 2004).

The mechanism of inhibition that phenolic compounds have against pathogens has more similarities with the action of vancomycin than that of tigecycline. Nevertheless, tigecycline combined with fermented cranberry juice displayed the most satisfying results against the pathogens tested.

Further investigation needs to be conducted in order to clarify the interaction between phenolic compounds from fruit extracts and antibiotics bioactive substances. A variety of functional foods, like beverages, could be produced and due to their synergistic or additive activity in combination with antibiotics, result in the application of such antibiotics in reduced doses against common pathogens.

## Conclusion

Functional beverages may include probiotic strains and provide specific health benefits beyond those of any normal food source. In our study, fermented cornelian cherry juice was examined against Gram-positive and Gram-negative pathogenic bacteria alone or in combination with vancomycin or tigecycline. Results clearly indicated a synergistic or additive interaction between cranberry juice and the antibiotic agent in most cases. Further experiments should be conducted for the clarification of this interaction.

## Data Availability

The raw data supporting the conclusions of this manuscript will be made available by the authors, without undue reservation, to any qualified researcher.

## Author Contributions

IM and EB were responsible for the conception of the study. IM was also responsible for the writing of the manuscript. ES isolated and characterized the pathogenic bacteria. CB, SP, ET, and SK were responsible for the susceptibility testing. AA and GD performed statistic analysis. CT and CV were responsible for the determination of the FICI.

## Conflict of Interest Statement

The authors declare that the research was conducted in the absence of any commercial or financial relationships that could be construed as a potential conflict of interest.
